# Mimickers of Large Vessel Giant Cell Arteritis

**DOI:** 10.3390/jcm11030495

**Published:** 2022-01-19

**Authors:** André Ramon, Hélène Greigert, Paul Ornetti, Bernard Bonnotte, Maxime Samson

**Affiliations:** 1 Rheumatology Department, Dijon-Bourgogne University Hospital, 21000 Dijon, France; paul.ornetti@chu-dijon.fr; 2 INSERM, EFS BFC, UMR 1098, RIGHT Graft-Host-Tumor Interactions/Cellular and Genetic Engineering, Bourgogne Franche-Comté University, 21000 Dijon, France; helene.greigert@chu-dijon.fr (H.G.); bernard.bonnotte@chu-dijon.fr (B.B.); maxime.samson@chu-dijon.fr (M.S.); 3 Internal Medicine and Clinical Immunology Department, Dijon-Bourgogne University Hospital, 21000 Dijon, France; 4 Vascular Medicine Department, Dijon-Bourgogne University Hospital, 21000 Dijon, France; 5 INSERM, CIC 1432, Clinical Investigation Center, Plurithematic Module, Technological Investigation Platform, Dijon-Bourgogne University Hospital, 21000 Dijon, France; 6 INSERM UMR 1093-CAPS, Bourgogne Franche-Comté University, UFR des Sciences et Du Sport, 21000 Dijon, France

**Keywords:** aortitis, large vessels vasculitis, giant cell arteritis, differentials diagnoses

## Abstract

Giant cell arteritis (GCA) is a large-vessel granulomatous vasculitis occurring in patients over 50-year-old. Diagnosis can be challenging because there is no specific biological test or other diagnoses to consider. Two main phenotypes of GCA are distinguished and can be associated. First, cranial GCA, whose diagnosis is usually confirmed by the evidence of a non-necrotizing granulomatous panarteritis on temporal artery biopsy. Second, large-vessel GCA, whose related symptoms are less specific (fever, asthenia, and weight loss) and for which other diagnoses must be implemented if there is neither cephalic GCA nor associated polymyalgia rheumatica (PMR) features chronic infection (tuberculosis, *Coxiella burnetti*), IgG4-related disease, Erdheim Chester disease, and other primary vasculitis (Behçet disease, relapsing polychondritis, or VEXAS syndrome). Herein, we propose a review of the main differential diagnoses to be considered regarding large vessel vasculitis.

## 1. Introduction

Giant cell arteritis (GCA) is the most common vasculitis in patients >50 years. It is a granulomatous vasculitis that affects large vessels (i.e., arteries outside the organs) [1], particularly the aorta and its extracranial branches (carotid, subclavian, axillary, vertebral, maxillary, occipital, and temporal arteries) [2].

Two phenotypes of GCA can be distinguished: 1—cranial GCA” that was first described by Bayard Horton in 1932 [3], with temporal headaches, jaw claudication, and scalp tenderness exposing patients to cranial ischemic manifestations (stroke, acute anterior ischemic optic neuropathy, occlusion of the central retinal artery). 2—”Large vessel (LV)-GCA” phenotype, which accounts for 30–70% of GCA patients. These two phenotypes can be associated, but some patients may have isolated involvement of the large arteries without cranial involvement. In this case, the diagnosis of GCA is more difficult [4] because symptoms are usually nonspecific and because other diagnoses have to be considered. Furthemore, these patients have an increased risk of aortic complications and/or cardiovascular events during the follow-up [5,6]. The risk of relapse also appears to be greater in this population, thus their identification is crucial [7].

The main manifestations of LV-GCA are constitutional symptoms (asthenia, anorexia, weight loss, unexplained fever) and nonspecific increase in acute phase reactants (erythrocyte sedimentation rate [ESR] and C-reactive protein [CRP]). Signs of vascular insufficiency are more common in LV-GCA than in cranial-GCA, such as asymmetric blood pressure, abolition of peripheral pulses, limb claudication, and/or ischemia [8].

**Figure 1:**
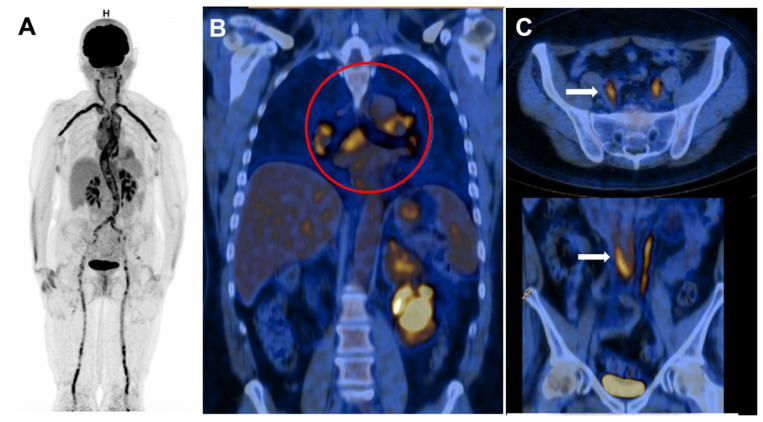
^18^FDG PET in giant cell arteritis (GCA) (**A**) and IgG4 related-disease (IgG4-RD) vasculitis (**B**,**C**). Panel A shows intense large vessel vasculitis of the thoracic and abdominal aorta, carotid arteries, subclavian arteries, iliac and femoral arteries in a patient with active GCA. Panel (**B**) shows mediastinal adenopathy (red circle) in IgG4-RD. Panel (**C**) shows tracer uptake in abdominal aorta and iliac arteries (white arrows) in IgG4-RD.

Currently, no specific diagnostic test is available to confirm LV-GCA since biopsy of these arteries is usually not performed in routine clinical practice except in the case of surgical intervention. LV-GCA is usually suspected by indirect evidence of large vessel vasculitis on vascular imaging such as an angio-CT scan, ^18^FDG PET, or angio-MRI. Aorta is the main site of inflammation, followed by the carotid and subclavian arteries [9] (Figure 1, panel A). Diagnostic of LV-GCA is highly suggested when large vessel vasculitis is associated with clinical or imaging PMR features [10].

Because of the lack of specificity of the initial clinical features and the absence of diagnostic tests for many conditions that can lead to inflammatory large vessel involvement (LV-GCA-like) (Table 1), the diagnosis of LV-GCA can be challenging and require that clinicians be very familiar with the differential diagnoses to be evoked.

In this review, the main differential diagnoses of LV-GCA are discussed.

## 2. Large Vessel Vasculitis

### 2.1. Infectious Aortitis

#### 2.1.1. Syphilis

Syphilis is a rare cause of aortitis. Most patients are asymptomatic, with an average age of 60 years at diagnosis [11]. It usually appears after 20 years of disease progression, in its tertiary phase [12]. In a series of 23 syphilitic patients, Roberts et al. [13,14] reported aneurysms in the ascending aorta (100%), the aortic arch (52%), and the descending thoracic aorta (43%). No involvement of the abdominal aorta was found. It is hypothesized that the absence of vasa vasorum in the abdominal aorta may explain the specific tropism of syphilis for the thoracic aorta [13]. In all cases, the aneurysm was fusiform. Histological analysis revealed thickening of the intima and the adventitia with an infiltrate of mononuclear cells and thickening/obliteration of the vasa vasorum. Media was not thickened but replaced by fibrous tissue with some mononuclear elements. Treatment relies on penicillin and surgical aneurysm resection as appropriate.

#### 2.1.2. Tuberculosis

Tuberculous aortitis is a rare condition. Aortic involvement is suspected in the presence of limb claudication, asymmetric blood pressure, and/or a decrease in peripheral pulses associated with pulmonary signs (nodular opacity, mediastinal adenopathy, pleural effusion) [15] or nonspecific signs such as an altered general condition, night sweats, and fever. The abdominal aorta is the most frequently involved (66% of cases) [12].

The classical features are pseudoaneurysms resulting from the hematogenous spread of *Mycobacterium tuberculosis* or damage to the vessel wall by contiguous adenitis. The evolution is generally favorable after anti-tuberculosis treatment, generally lasting for 12 to 18 months.

#### 2.1.3. *Coxiella burnetti*

*Coxiella burnetti* can cause aortitis. Some cases of Q fever mimicking LV-GCA have been reported [16,17,18]. In a retrospective French multicenter study, 8/55 aortitis revealed *Coxiella burnetti* infection. Main aortitis localization was abdominal (54%) and thoracic (33%). Fusiform aneurysm was the main aortitis pattern [19]. The diagnosis is difficult because the positive serology may be related to an old infection, unrelated to the aortic involvement. The study of antibody avidity can help date the infection. A low avidity indicates a recent infection. The diagnosis of certainty is provided by demonstrating the bacterium by culture, PCR, or immunohistochemistry on vascular or prosthetic tissue or a periarterial abscess or spondylodiscitis in case of contiguous involvement near the aorta. Treatments rely on a combination of hydroxychloroquine (600 mg once a day) and doxycycline (100 mg twice a day) for 24 months.

#### 2.1.4. Other Infectious Causes

Many bacterial germs can cause aortitis. The most common are gram-positive cocci, *Staphylococcus aureus,* and *Streptococcus*, the latter often being implicated in the occurrence of aortic aneurysm in the context of infectious endocarditis [20,21,22]. Cases of *Salmonella* aortitis have also been reported, mainly in the abdominal aorta [23,24].

Other germs have been reported to trigger aortitis: *Listeria monocytogenes* [25], *Pasteurella multicoda* [26], and *Clostridium septicum* [27].

The diagnosis is usually suspected based on the clinical presentation, the patient’s medical history (immunosuppression), and the circumstances of occurrence (bite). Gram-negative bacillus involvement may reveal associated malignancy [28].

#### 2.1.5. Severe Acute Respiratory Syndrome Coronavirus-2 (SARS-CoV-2)

During the COVID-19 pandemic, Lecler et al. reported a 70% increase in GCA cases at their center, raising the question of a direct role of SARS-CoV-2 in the occurrence of GCA [29].

Cases of aortitis attributed to SARS-CoV-2 have also been reported [30,31]. Silvestri et al. [31] reported 17 cases of aortic involvement during SARS-CoV-2 infection. Mean age at diagnosis was 58.6 +/− 15.2 years, 70.5% of described patients were men and 64.7% of had cardio-vascular comorbidities (mainly hypertension [47%], renal pathology [17.6%], coronary artery disease ([17.6%], previous aortic surgery [11.7%] and arrhythmia [11.7%]). Main clinic-biological features were fever (47%), chest pain (47%), respiratory symptoms (35.2%), and lymphopenia (17.6%). Aortic pathology included: type A aortic dissection (64.7%), new pathology of previous aortic graft (11.7%), aortitis (11.7%), thoracoabdominal aortic aneurysm (5.9%), one ruptured aortic aneurysm (5.9%), and one aortic embolizing thrombosis (5.9%). Open surgery or endovascular treatment were carried out in 58.8 and 17.6% of cases, respectively. Four patients (23.5%) died, three before surgery.

SARS-CoV-2-induced aortitis is thought to be an infectious aortitis occurring during the viremic phase. During this phase, the virions would directly attack the vascular endothelium, which highly expressed angiotensin-converting enzyme-2 receptors, leading to arterial inflammatory lesions [32]. Large vessel involvement during SARS-CoV-2 infection could be due to a direct effect of the virus on the endothelium leading to endothelial dysfunction and recruitment of inflammatory cells. Manenti et al. [33] hypothesized that atherosclerosis and, more particularly, the presence of ulcerated plaques, which is common in elderly subjects, could be a facilitating factor. However, there are currently too few cases to accurately study the pathogenesis of these vasculitis.

Some cases of GCA following SARS-CoV-2 vaccination have also been described. Using VigiBase analysis (26,246,383 reports until 30 June 2021 with 1,295,482 reports concerning COVID-19 vaccines), Mettler et al. [34] reported 147 cases of GCA, 290 cases of polymyalgia rheumatica (PMR) and 9 GCA with PMR cases, which resulted in an increased risk of GCA (ROR [reporting odds ratio] = 2.7 [95% CI: 2.3; 3.2]) and PMR (ROR = 2.3 [95% CI: 2.0; 2.6]) following SARS-CoV-2 vaccination, whereas it was not the case after influenza vaccination for GCA (ROR = 0.5; [95% CI: 0.4; 0.7]) and PMR (ROR = 0.2; [95% CI: 0.2; 0.2]). Median (IQR) time from vaccination to first symptoms onset was 4 (1–14) days. Cases reported with mRNA vaccine were 61.9% of the total and with viral vector vaccine 37.4%. Fifteen percent of GCA patients had ophthalmological symptoms. No details about large vessel involvement are provided in this study.

Sauret et al. [35] also reported the case of a patient who developed GCA following SARS-CoV-2 vaccination. The patient was positive for HLA-DR4, which made the author hypothesize that HLA could play a role in the occurrence of post-vaccinal GCA.

### 2.2. IgG4 Related Disease (IgG4-RD)

IgG4-RD is a fibro-inflammatory condition characterized by an IgG4-rich lymphoplasmacytic infiltrate. It is a multi-systemic disease involving the biliary tree, salivary glands, lungs, periorbital tissues, kidneys, meninges, prostate, pericardium, skin, retroperitoneum, and aorta [36]. Lymph nodes enlargement is reported in 14% of IgG4-RD patients [37] (Figure 1, panel B)

The diagnosis is suspected in cases of elevated serum IgG4 level (>1.35 g/L). However, isolated elevated serum IgG4 can be observed in pancreatic cancer and the normal population [38]. Thus, histopathological analysis is the cornerstone of the diagnosis. The most specific histological lesions are a lympho-plasmacytic infiltrate associated with storiform fibrosis, obliterating thrombosis, and a small to moderate eosinophilic infiltrate [39]. Even if the IgG4 infiltrate is not specific to the disease, an IgG4 bearing plasma cell to IgG bearing plasma cell ratio >50% in affected tissues is very suggestive for diagnosing IgG4-RD [36].

Aortic involvement is variable in IgG4-RD (10–50% of cases) [40,41]. It is characterized by peri-aortitis (20 to 36%) that more frequently affects the sub-renal aorta and aortitis (Figure 1, panel C) (8%) that more frequently affects the thoracic aorta and leads to the occurrence of inflammatory aneurysms. Retroperitoneal fibrosis is found in 3 to 19% of patients and can cause obstructive renal failure. Isolated aortic involvement is rare because 80% of patients have associated symptoms of IgG4-RD [42].

Acute phase reactants are usually moderately increased. From 89 IgG4-RD with large vessels vasculitis, Peng et al. reported a mean ESR value of 44 mm/h (18–75) and mean usCRP of 6.72 mg/L (2.14–24.65) [43]

Wallace et al. [44] identified 4 patient phenotypes: pancreato-hepatobiliary, head and neck, aortitis/retroperitoneal fibrosis, and Mikulicz disease. The treatment of IgG4-RD relies on systemic glucocorticoids. The disease is very sensitive to glucocorticoids but tends to relapse when the doses are decreased. Rituximab is, therefore, very useful in this indication [45].

### 2.3. Behcet Disease (BD)

Aortic involvement in BD occurs in 4–34% of cases [46,47]. The most frequent lesions are aneurysms (70%) of the abdominal (11%) and thoracic (5%) aorta. Diffuse aortitis is rarer (3%) [46].

Suggestive features of the disease are mainly mouth and genital ulcers (63 to 100%), skin involvement (68%), ophthalmological disorders (48%), and joint manifestations (38%) [46].

Features associated with aortic involvement include abdominal or lumbar pain, limb claudication, and aortic insufficiency. Fever is present in 11% of cases. [48]. Arterial involvement management relies on glucocorticoids or other immunosuppressive drugs (azathioprine, cyclophosphamide, cyclosporine A). Monoclonal anti-TNF antibodies can be considered in refractory cases. In the case of artery aneurysms, cyclophosphamide and corticosteroids are required before surgical intervention or stenting [49,50].

Survival rates for patients with aortic involvement are significantly lower than patients without aortic involvement [46].

### 2.4. Erdheim Chester Disease (ECD)

ECD is a histiocytosis of the “L” group [51] characterized by the presence of foamy histiocytes CD68^+^CD163^+^FXIIa^+^CD1a^−^. More than 80% of patients have an activating mutation of the MAPK (Mitogen-Activated Protein Kinase) pathway, mainly BRAF ^V600E^ (57% to 70%) [52].

**Figure 2:**
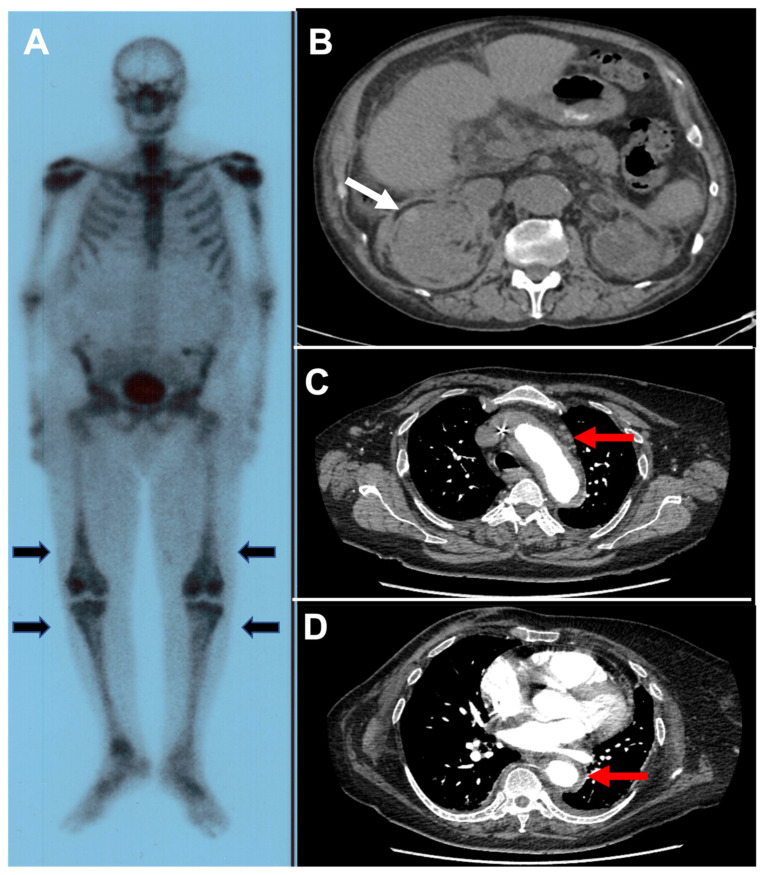
Erdheim Chester disease (ECD). Panel (**A**): Uptake of the tracer by long bones on the ^99^Technecium bone scintigraphy (black arrows). Panel (**B**): CT-scan showing a symmetrical infiltration of the perirenal fat and of the perirenal fascia taking the appearance of “hairy kidneys” (white arrow). Panel (**C**,**D**): angio-CT scan showing a peri-aortitis (coated aorta) in a patient with ECD (red arrows).

ECD predominantly affects adult males with a median age of 55 years at diagnosis. The usual features of this multi-systemic disease include cardiac involvement (right atrium pseudotumor, coronary infiltration, pericarditis), xanthelasma, long bone osteosclerosis (Figure 2, panel A), diabetes insipidus, central nervous system involvement, renal involvement (hairy kidneys) (Figure 2, panel B), retroperitoneal fibrosis, pulmonary involvement (interstitial lung disease, pleural infiltration) [53].

Aortic involvement is reported in 40% to 60% of cases [52,54], in the form of a peri-aortic infiltrate (coated aorta) (Figure 2, panel C,D) or ectasia affecting the thoracic and abdominal aorta, which may extend to the main branches of the aorta. It rarely affects the pelvic and lower limb arteries. This aortitis is usually asymptomatic. Treatment relies on pegylated interferon-α (IFN-α) as first line therapy. Second line therapies include BRAF inhibitors (vemurafenib) or MEK inhibitors (cobimetinib) according to mutation profile [53].

### 2.5. Iatrogenic Cause

#### 2.5.1. Immune Checkpoint Inhibitors (ICI)

Few cases of GCA secondary to the introduction of ICI (anti PD-1/PD-L1, anti CTLA-4) therapy have been reported even though PD-1/PD-L1 pathway dysfunction has been demonstrated during GCA [55].

Among the few cases reported [56,57], patients were mostly treated with anti PD-1 antibodies for metastatic melanoma. Ophthalmologic signs and PMR were mentioned in two and one case, respectively.

In a retrospective study assessing cardiovascular toxicity of ICI, authors reported 18 cases of temporal GCA, with a clear male predominance (94.5%) and a mean age at diagnosis of 77.8 years. More than half of the cases occurred following an anti CTLA-4 ICI (55%). Only 16.7% of patients had headaches, and 27% had visual signs [58].

#### 2.5.2. Granulocyte Colony-Stimulating Factor (G-CSF)

G-CSF is a human granulocyte hematopoietic growth factor. Recombinant G-CSF is used as a primary or secondary prophylaxis to reduce the risk of neutropenia secondary to myelosuppressive cytotoxic chemotherapy [59]. There are several reports of aortitis in patients treated with recombinant G-CSF [60,61,62]. Oshima et al. [63], based on 3409 patients treated with recombinant G-CSF, reported a significant association between aortitis and the use of G-CSF (odds ratio [OR] = 45.87; *p* < 0.001).

In most cases, patients have recovered within 1 month after systemic glucocorticoid therapy and discontinuation of G-CSF [60,61,62].

### 2.6. Large Vessel Vasculitis Associated with Inflammatory Rheumatisms

A few cases of aortitis have been reported in rheumatoid arthritis. Factors associated with aortitis include severe arthritis, duration of disease >1 year, the presence of erosions, and rheumatoid nodules. The presence of HLA-DRB1*0401 has been reported in the old series as associated with the occurrence of rheumatoid vasculitis [64].

Aortitis has also been reported in spondyloarthritis (ankylosing spondylitis and psoriatic arthritis) [65]. Involvement of the ascending aorta and the aortic arch is the most common; involvement of the abdominal aorta is rarer [66].

Aortic involvement has also been reported in relapsing polychondritis (RP) [67,68,69,70]. Involvement of the ascending aorta and the aortic arch is the most common. Le Besnerais et al. [70] reported aortic involvement in 11/172 patients with RP (aortitis [18%], isolated aneurysm [36%], aortitis + aneurysm [18%]). The mean time to diagnose aortic involvement was 27 months after the diagnosis of RP. More than half of the patients were male, had fever and asthenia. Aortic involvement is associated with a high mortality rate in this population (23–27%).

Some cases of large vessel involvement have been reported in sarcoidosis. The diagnosis can be very challenging in the case of sarcoidosis, especially since sarcoidosis can result in rheumatologic involvement such as PMR [71]. Histological analysis of affected tissues, vascular or not, shows the presence of giganto-cellular granulomas without necrosis. Treatment relies on systemic glucocorticoid therapy [72].

Similarly, cases of aortitis have been reported in systemic lupus erythematosus (SLE) [73]. At diagnosis, clinical signs are nonspecific (fever, dyspnea, and chest pain), and inflammatory markers (CRP and ESR) are elevated. Aortitis mainly affected the ascending aorta with circumferential thickening (60%) or inflammatory aneurysm (20%). Most of the patients had isolated aortitis without another clinical sign of SLE. All patients showed a high titer of anti-nuclear antibodies with anti-double-stranded DNA and/or anti smith antibodies.

A new disease based on clonal hematopoiesis, named VEXAS (Vacuoles, E1 enzyme, X-linked, autoinflammatory, somatic), has been identified recently [74]. VEXAS syndrome is related to somatic mutations in the *UBA1* gene occurring in myeloid cells. The frequency of this disease is probably underestimated, and its precise clinical features are largely unknown. Patients are men with recurrent fever, dermatologic manifestations (neutrophilic dermatoses and cutaneous vasculitis), pulmonary infiltrate, ear and nose chondritis, macrocytic anemia, and hematopoietic dyspoiesis. Some patients also have large vessel vasculitis. The disease is sensitive to high-dose glucocorticoids, but patients are generally dependent on high doses and therefore quickly require the use of other therapies, including tocilizumab, 5-azacitidine, or ruxolitinib [75,76]. VEXAS syndrome should be discussed in male patients with atypical features of LV-GCA together with hematologic abnormalities (macrocytic anemia or myelodysplastic syndrome), high-dose corticodependence, and/or overlapping with RP or neutrophilic dermatoses [77].

### 2.7. Atherosclerosis

Atherosclerosis may raise suspicion of LV-GCA, but some characteristics usually differentiate these two conditions.

CRP is usually not significantly elevated (i.e., <10 mg/L) in atherosclerosis, by contrast with LV-GCA [78,79]. The topography of the lesions can also guide the clinician. Atheromatous lesions are most often located in the vessels of the lower limbs and form focal lesions at the origin of the collaterals. By contrast, GCA lesions are diffuse and more commonly involve the ascending aorta, supra-aortic trunks, and the aortic arch [80].

When performing ^18^FDG PET, the intensity of FDG tracer uptake helps in distinguishing these two diagnoses. The uptake is generally lower in atherosclerosis (grade 1) than in GCA (grade 2 or 3). In addition, atherosclerosis lesions have a more heterogeneous and irregular appearance, whereas LV-GCA lesions are more homogeneous and linear [81,82].

### 2.8. Malignancy

Features of LV-GCA (weight loss, asthenia, anorexia, fever, biological inflammatory syndrome) may raise suspicion of malignancy.

According to some studies, associations between GCA and malignancy are reported in 10–25% of patients [83,84] with a relative risk (RR) of 2.16 within 6 months after the diagnosis of GCA [85]. A retrospective study reported a higher risk of solid malignancy (Hazard Ratio (HR) = 1.2) and hematological malignancy in GCA compared to a control population. Higher age at diagnosis and male gender were associated with malignancy [86]. By contrast, other studies did not confirm these results [87].

Beyond this possible association between GCA and malignancy, some cases of paraneoplastic GCA are reported [88], notably during myelodysplastic syndrome [89,90], but these cases have been reported before the first description of VEXAS syndrome.

### 2.9. Associated Features of PMR

PMR is an inflammatory disorder with severe pain and stiffness of the scapular and pelvic girdle occurring in patients over 50 years associated with increased acute phase reactants (ESR, CRP) [10]. It is associated with GCA in 16 to 21% of cases, and 40 to 60% of patients with GCA have associated features of PMR [91]. The presence of PMR is therefore a feature that is often very suggestive of GCA in case of large vessel vasculitis without cranial signs of GCA.

However, inflammatory girdle pain may indicate other and sometimes more severe conditions that warrant specific diagnostic and therapeutic management. Many diseases can mimic PMR (PMR-like) and need to be ruled out before confirming the diagnosis of PMR, especially as some may be accompanied by large vessel vasculitis as elderly onset rheumatoid arthritis (EORA) and spondyloarthritis.

EORA can mimic PMR by involving the large proximal joints, usually with a sudden onset, associated with general signs and a male predominance [92]. A key difference between EORA and PMR is the presence of the rheumatoid factor (RF) and anti-citrullinated peptide antibodies (ACPA) in EORA. Moreover, Pease et al. reported that RF-negative EORA was more frequently associated with peripheral small joint involvement (metacarpophalangeal and proximal interphalangeal joint) (80% vs. 23% in PMR). Similarly, in a small series of patients (15 PMR and 7 EORA), Wakura et al. [93] demonstrated, using ^18^FDG PET of 9 anatomical sites, that PMR patients had more frequent fixation abnormalities than EORA subjects. The main differences between the two groups were periarticular hyperfixation of the shoulder girdle, the coxofemoral joint, the pectineal muscle, and the ischial tuberosity entheses, as well as hyperfixation of the spinous processes in the cervical and lumbar regions and posterior joint involvement in the lumbar region in favor of the PMR group.

Late-onset spondyloarthritis (LOPS) may involve the cervical spine and shoulder girdle with constitutional manifestations as low-grade fever, asthenia, and weight loss in association with increased acute phase reactants [94]. Radiographic sacroiliitis and HLA-B27 positivity are key elements for diagnosis.

## 3. Conclusions

Isolated LV-GCA remains a diagnostic challenge for the clinician, as many conditions may be responsible for large vessel involvement. Its early diagnosis is important because of the risk of arterial complications in the medium and long term that can compromise a patient’s survival. The diagnosis is based on the association of clinical (associated PMR symptoms) and biological and imaging features and requires ruling various conditions, particularly infectious and other inflammatory diseases.

In the absence of specific disease markers, the clinician must be aware of the numerous differential diagnoses to offer patients early and appropriate management.

## Figures and Tables

**Table 1:** Differential diagnoses of “large vessel”-giant cell arteritis: Infectious and systemic disease.

	Clinic	Laboratory Findings
IgG4-related disease	Retroperitoneal fibrosis ***Lymphatic involvement:*** supra-centimetric adenopathy ***Digestive disorders*:** pancreatitis, steroid-sensible cholangitis ***Ophthalmological involvement***: Dacryocystitis, dacryoadenitis, orbital pseudotumor ***ENT involvement***: sialadenitis, parotid hypertrophy ***Neurological involvement***: headache, cranial nerve paralysis, radiculopathy, pachymeningitis ***Endocrine disorders***: diabetes, hypothyroidism ***Pulmonary involvement***: pleural effusion, diffuse interstitial lung disease ***Aortic disease***: aortic dissection, aneurysm, periaortitis, aortitis	Elevated serum IgG4 (>1.35 g/L) (80%) Increased ESR and CRP Hypereosinophilia Polyclonal hypergammaglobulinemia
Erdheim Chester disease	***Cardiac involvement***: pericarditis, right atrial pseudotumor, coronary infiltration ***Pulmonary involvement***: interstitial lung disease, pleural infiltration ***Arterial disease***: coated aorta ***Bone damage***: osteosclerosis of long bones, bone pain ***Skin involvement***: periorbital xanthelasma, papulo-nodular rash ***Renal involvement***: hydronephrosis, hairy kidneys, retroperitoneal fibrosis ***Endocrinological disorders***: diabetes insipidus, growth hormone deficiency, hyperprolactinemia ***Neurological impairment***: pyramidal syndrome, cerebellar syndrome, epilepsy, headache, cognitive disorders	Foamy histiocytes CD68^+^CD163^+^FXIIa^+^CD1a^-^ BRAF^V600E^ (57% to 70%) Increased ESR and CRP
Infection	Fever, Altered condition Sepsis, Heart murmur ***Patient’s medical past history:*** Immunosuppression IV addiction	Increased ESR and CRP Bacteriological findings
Behcet	***Skin involvement***: mouth and genital ulcers, pseudofolliculitis, erythema nodosum ***Ophthalmological involvement***: anterior uveitis with hypopyon, posterior uveitis (occlusive and necrotizing vasculitis) ***Joint disorders***: arthralgia, oligoarthritis (knees, ankles) ***Arterial diseases***: aortitis, aneurysm ***Venous disease***: superficial and deep vein thrombosis ***Neurological involvement***: headache, meningoencephalitis, cranial nerve paralysis, pyramidal signs	HLA B51 Increased ESR and CRP Pathergy test
Rheumatoid Arthritis	Bilateral and symmetrical destructive polyarthritis ***Extra-articular involvement***: rheumatoid nodule, diffuse interstitial lung disease, rheumatoid pleuritis, scleritis, episcleritis	Increased ESR and CRP Positive RF and ACPA.
SpA	Inflammatory spinal pain/asymmetric oligoarthritis, tilted pygalia ***Extra-articular manifestations***: cutaneous psoriasis, non-granulomatous anterior tilt uveitis Chronic inflammatory bowel disease (Crohn’s disease, hemorrhagic rectocolitis)	Positive HLA-B27 (50 to 90%) Increased ESR and CRP
Relapsing polychondritis	***Chondritis***: ear lobe, nasal, respiratory tree, costal cartilages ***General signs***: fever, asthenia, weight loss ***Joint manifestations***: arthralgias, oligoarthritis, asymmetric non-erosive, migratory polyarthritis ENT manifestations: sensorineural hearing loss ***Ophthalmological manifestations***: scleritis, episcleritis, conjunctivitis ***Skin manifestations***: vascular purpura, ring urticaria	Increased ESR and CRP Positive RF (15%) Anti-collagen type 2 antibodies (lack of specificity) Association with myelodysplastic syndrome
Systemic lupus erythematous	***Skin involvement****:* malar rash, discoid rash, photosensitivity ***Neurological involvement****:* seizure, psychosis ***Joint involvement****:* non-erosive arthritis ***Renal involvement****:* glomerulonephritis pleuritis, pericarditis	Increased ESR and moderate increase in CRP Positivity of anti-nuclear antibody (anti-double-stranded DNA, anti-smith antibodies) Decreased C3 Antiphospholipid antibodies Hemolytic anemia, lymphopenia, thrombocytopenia Renal failure, proteinuria, hematuria
Sarcoidosis	***General signs***: fever, asthenia, weight loss ***Skin involvement****:* dermic sarcoid, erythema nodosa ***Pulmonary involvement****:* mediastinal lymph nodes, interstitial lung disease ***Ophthalmological manifestations:*** granulomatous anterior uveitis, posterior uveitis ***Joint disorders***: arthralgia, migratory arthritis (ankle++) ***Neurological involvement****:* headaches, cognitive/behavioral disorders, seizures, cranial nerve paralysis ***Cardiac involvement***: arrhythmias, conduction defects, sudden cardiac death ***Renal involvement:*** interstitial nephritis, lithiasis and renal calcinosis	Lymphopenia (CD4 T cells) Increased ESR and CRP Increased angiotensin-converting enzyme Polyclonal hypergammaglobulinemia Hypercalcemia, hypercalciuria Increase 1,25 (OH)2 vitamin D Histology: giganto-cellular granuloma without necrosis
VEXAS syndrome	***Fever*** ***Skin involvement****:* neutrophilic dermatoses (Sweet syndrome), leukocytoclastic vasculitis, medium-sized vasculitis ***Lung involvement***: pulmonary infiltrate, pleural effusions ***Ear and nose chondritis*** ***Venous thromboembolism*** ***Arthritis*** ***Large-vessel vasculitis*** ***Venous thromboembolism*** ***Orchitis/epididymitis***	Increased ESR and CRP Somatic UBA1 variant (p.Met41) Features of myelodysplastic syndrome: Macrocytic anemia Thrombocytopenia; bone marrow vacuoles (restricted to myeloid and erythroid precursor cells)

SpA: spondyloarthritis, RF: rheumatoid factor, ACPA: anti-citrullinated peptide antibodies, ESR: erythrocyte sedimentation rate, CRP: C-reactive protein., VEXAS: vacuoles, E1 enzyme, X-linked, auto-inflammatory, somatic.

## Data Availability

Not applicable.
